# Hemorrhage from Extra-Antral Gastric Antral Vascular Ectasia in a Patient with Duodenal Heterotopic Gastric Mucosa

**DOI:** 10.1155/2016/4325302

**Published:** 2016-10-18

**Authors:** John Gubatan, Nathan Raines, Hasan Khosravi, Tracy L. Challies, Tyler M. Berzin

**Affiliations:** ^1^Department of Medicine, Beth Israel Deaconess Medical Center, Harvard Medical School, Boston, MA, USA; ^2^Department of Pathology, Beth Israel Deaconess Medical Center, Harvard Medical School, Boston, MA, USA; ^3^Division of Gastroenterology and Hepatology, Beth Israel Deaconess Medical Center, Harvard Medical School, Boston, MA, USA

## Abstract

Gastric antral vascular ectasias (GAVE) have been increasingly recognized as an uncommon cause of chronic gastrointestinal bleeding and anemia, although their underlying pathogenesis is not completely well understood. Heterotopic gastric mucosa (HGM) has been reported to occur at various sites along the gastrointestinal tract and although relatively common, it is often asymptomatic. We report a case of a 60-year-old woman with a prior history of GAVE who developed melena and symptomatic anemia during her hospitalization following cardiac catheterization. Initial EGD demonstrated nonbleeding antral GAVE and a newly discovered duodenal mass. Duodenal mass biopsies were ultimately notable for HGM along with histologic features of extra-antral GAVE. The patient required blood transfusions and consequently had a small bowel endoscopy notable for fresh blood in the proximal small bowel. The patient underwent a small bowel push enteroscopy which demonstrated active bleeding of the duodenal mass and overlying oozing GAVE, which was cauterized with Argon-Plasma Coagulation with adequate hemostasis. We present for the first time a novel association between GAVE and HGM. Our case illustrates that extra-antral GAVE may occur with HGM in the duodenum. We explore potential mechanisms by which HGM may be involved in the pathogenesis of GAVE.

## 1. Introduction

Gastric antral vascular ectasia (GAVE) is a rare disorder characterized by its distinctive endoscopic appearance consisting of red, tortuous ectatic blood vessels arranged in a longitudinal array localized in the gastric antrum converging on the pylorus [1]. Prominent histological features of GAVE include fibromuscular hyperplasia of the lamina propria, capillary ectasia, presence of intravascular fibrin thrombi, and mucosal architectural distortion [2]. GAVE occurs in about a third of patients with cirrhosis and portal hypertension [3]. In noncirrhotic patients, GAVE has been associated with autoimmune disorders, connective tissue disorders, chronic kidney disease, and ectopic pancreas [4–7]. Although GAVE is rare, it accounts for up to 4% of nonvariceal upper gastrointestinal bleeding [8]. Heterotopic gastric mucosa (HGM), gastric tissue found outside the stomach, is found to occur anywhere along the gastrointestinal tract and has been estimated to have a prevalence of 0.1% to 3% [9]. HGM is often asymptomatic and found incidentally on endoscopic studies. In particular, duodenal HGM has been shown to not be associated with significant GI symptoms in previous studies [10, 11]. Although previous cases of duodenal HGM have been described, we report for the first time an unusual case of a patient with known GAVE who presented with melena and was found to have a bleeding duodenal mass with histology consistent with HGM and features of extra-antral GAVE.

## 2. Case Presentation

A 60-year-old woman with a prior history of GAVE was referred from an outside hospital with exertional dyspnea and further workup of echocardiogram abnormalities. The patient's medical history was most notable for hypertension, diabetes mellitus, coronary artery disease, chronic obstructive pulmonary disease, and diastolic heart failure, but no history of known cirrhosis, connective tissue disorder, or autoimmune disease. The patient's GAVE was diagnosed one year prior to presentation. At that time, she presented with melena that required intermittent blood transfusions for symptomatic anemia. She had an esophagogastroduodenoscopy (EGD) which was notable for GAVE. Argon-Plasma Coagulation (APC) was applied to these angioectasias with successful hemostasis.

On admission, the patient was hemodynamically stable, hemoglobin and hematocrit were at baseline, and the patient denied melena. The patient's echocardiogram demonstrated inferoposterolateral wall hypokinesis. The patient subsequently underwent left cardiac catheterization which demonstrated a lesion along the right marginal artery which was stented. The catheterization was complicated by a right coronary artery dissection and ST elevation myocardial infarction (STEMI) requiring three drug-eluting stents. The patient was started on dual antiplatelet therapy with Ticagrelor and aspirin and managed in the cardiac intensive care unit (CCU). In the CCU, the patient experienced significant melena with drop in hemoglobin (from 8.5 g/dL to 6.0 g/dL), requiring blood transfusions. Abdominal CT did not demonstrate a retroperitoneal hematoma or any other source of bleeding. The patient remained hemodynamically stable and hemoglobin increased appropriately with blood transfusions.

**Figure 1:**
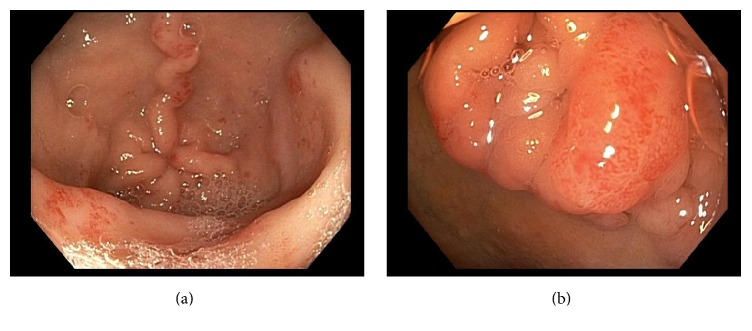
Initial esophagogastroduodenoscopy (EGD) demonstrating gastric antral vascular ectasias (GAVE) without evidence of active oozing (a) and frond-like mass in the duodenal bulb with no active bleeding or stigmata of recent bleed (b).

**Figure 2:**
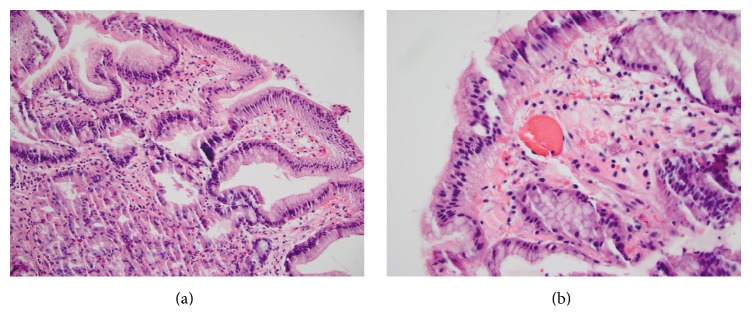
Duodenal biopsies demonstrating oxyntic gastric glandular cells (specialized acid secreting) underlying gastric foveolar epithelium on the surface, no goblet cells or enterocytes to identify it as intestinal (a), and presence of dilated and ectatic blood vessels with mild vascular congestion, features characteristic of GAVE (b).

The patient underwent further evaluation with colonoscopy and EGD. Colonoscopy was unremarkable. EGD demonstrated small angioectasias in the antrum consistent with known GAVE without evidence of oozing (Figure 1(a)). EGD was most notable for a 2 cm frond-like mass in the duodenal bulb with no active bleeding (Figure 1(b)). Biopsies were obtained from this duodenal mass with histology consistent with heterotopic gastric mucosa (Figure 2(a)) and dilated and ectatic blood vessels with mild vascular congestion, features suggestive of GAVE. On histology, there was no evidence of atrophic gastritis and* Helicobacter pylori* staining was negative. The patient was managed on an oral proton pump inhibitor twice daily. On the medical floor, the patient continued to experience intermittent exertional dyspnea and chest pain. The patient was managed with intravenous diuretics for diastolic heart failure exacerbation and started on oral nitrates for anginal pain from her recent STEMI with improvement in her symptoms. The patient continued to experience melena with worsening anemia and consequently required intermittent blood transfusions.

**Figure 3:**
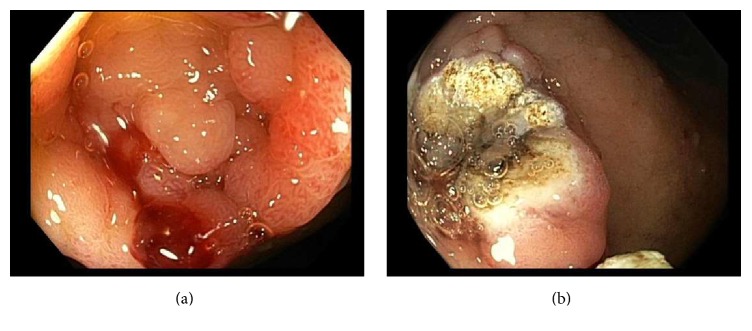
Small bowel push enteroscopy: gastric heterotopia in duodenal bulb with overlying oozing extra-antral GAVE (a) and duodenal mass after Argon-Plasma Coagulation (APC) was applied for successful hemostasis (b).

In the setting of continued gastrointestinal bleeding, the patient had a capsule endoscopy which demonstrated fresh blood in the duodenum and proximal small bowel with no clear source of bleeding. The patient subsequently underwent a small bowel push enteroscopy which demonstrated bleeding from the previously visualized duodenal mass, with overlying oozing extra-antral GAVE (Figure 3(a)). The duodenal mass composed of gastric heterotopia was cauterized with Argon-Plasma Coagulation with successful hemostasis (Figure 3(b)). After the endoscopic procedure, the patient had less episodes of melena and repeat hemoglobin/hematocrit levels were robustly stable. She was discharged home with close follow-up with gastroenterology and plan for regular surveillance of her hemoglobin.

## 3. Discussion

In the current case, we present a patient with a history of GAVE who presented with melena and symptomatic anemia and was not found to have bleeding GAVE as expected but rather duodenal HGM with histologic features of extra-antral GAVE as an etiology of the patient's gastrointestinal bleeding. The patient was successfully treated with a proton pump inhibitor, blood transfusions, and endoscopic APC to duodenal mass.

Most cases of gastric antral vascular ectasia (GAVE) have been reported to occur in the gastric antrum. One study by Stotzer et al. [12] reported that ectatic lesions are also common in the gastric cardia. However, GAVE has not previously been reported to occur in the duodenum. This is the first reported case in the literature to demonstrate that GAVE may occur in the duodenum with associated HGM. Our finding challenges the paradigm that GAVE only occurs within the gastric antrum and raises some interesting aspects regarding the pathogenesis of GAVE.

The temporality between GAVE and duodenal HGM in our patient is an interesting point. The patient had GAVE visualized by endoscopy one year prior to her current presentation. At that time, the 2 cm duodenal mass was not seen. Heterotopic gastric mucosa in the duodenum is usually congenital, representing a developmental anomaly [11]. The duodenal mass was unlikely from metaplastic change as the patient had no chronic inflammation or peptic ulcer disease in the surrounding area. The patient's duodenal HGM was likely congenital and may have been missed by endoscopic visualization during her last EGD. The fact that the patient's duodenal HGM likely preceded her antral GAVE raises the possibility that HGM may be a risk factor for the development of GAVE. Although ectopic pancreas has been previously described in a patient with associated GAVE [7], heterotopic gastric mucosa in a patient with GAVE has not been reported in the literature.

One hypothesis is that GAVE arises from mechanical stress due to increased gastric peristalsis. Charneau et al. demonstrated [13] that patients with GAVE had abnormal antral motility. Demonstrating extra-antral GAVE in the duodenum suggests that gastric peristalsis and abnormal antral motility are not necessary in the development of GAVE. However, mechanical stress may still play a role in the formation of GAVE as duodenal contractions do occur, although they differ from antral contractions with regard to amplitude and frequency [14]. It is unclear if duodenal motility plays a role in the development of extra-antral duodenal GAVE.

Hypergastrinemia has been previously implicated in the pathogenesis of GAVE [15]. One study demonstrated that patients with gastric oxyntic heterotopia found in the duodenum had increased serum gastrin levels [16]. Our patient's duodenal mass histology indeed demonstrated oxyntic gastric glandular cells. Although we did not measure serum gastrin in our patient, it is possible that our patient also had elevated serum gastrin levels. Hypergastrinemia may have predisposed our patient to developing antral GAVE and extra-antral GAVE in the setting of heterotopic gastric mucosa.

It is interesting to note that GAVE has been described in patients with atrophic gastritis [17, 18]. Patients with atrophic gastritis are known to develop hypergastrinemia. Destruction of parietal cells in atrophic gastritis leads to profound hypochlorhydria which induces G-cell (gastrin-producing) hyperplasia and consequently hypergastrinemia [19, 20]. Hypergastrinemia from underlying atrophic gastritis may be a possible mechanistic link in the association of GAVE with atrophic gastritis. Histology of the duodenal HGM mass in our patient did not demonstrate any features of atrophic gastritis and was negative for* Helicobacter pylori* on staining. Although we did not obtain gastric mucosal biopsies for histologic evaluation, clinical suspicion in our patient for atrophic gastritis was low. Our patient did not have any symptoms (nausea, vomiting, anorexia, weight loss, and abdominal pain) and labs were not suggestive of iron deficiency or megaloblastic anemia. Thus, atrophic gastritis as a mechanism for the development of GAVE in our patient was unlikely.

Another potential mechanism in the pathogenesis of GAVE involves the local proliferation of neuroendocrine cells which in turn produce high levels of vasoactive substances such as vasoactive intestinal polypeptide and serotonin which result in vascular dilation [21]. Neuroendocrine cells were not visualized on histology from our duodenal specimen; thus this particular pathogenic mechanism is unlikely in our patient.

In conclusion, heterotopic gastric mucosa is associated with the development of GAVE, possibly through increased serum gastrin production owing to ectopic gastric mucosa. Our case report suggests that HGM may be associated with GAVE and that extra-antral GAVE may develop beyond the gastric antrum. Bleeding extra-antral GAVE in the duodenum appears to be responsive to proton pump inhibitor and endoscopic thermal ablation therapy.

## Abbreviations

GAVE: Gastric antral vascular ectasia
HGM: Heterotopic gastric mucosa
EGD: Esophagogastroduodenoscopy
APC: Argon-Plasma Coagulation
STEMI: ST elevation myocardial infarction
CCU: Cardiac intensive care unit
CT: Computed tomography.
